# The mechanism of exercise for pain management in Parkinson’s disease

**DOI:** 10.3389/fnmol.2022.1039302

**Published:** 2022-11-10

**Authors:** Wen-Ye Yu, Qi-Hao Yang, Xue-Qiang Wang

**Affiliations:** ^1^Department of Sport Rehabilitation, Shanghai University of Sport, Shanghai, China; ^2^Department of Rehabilitation Medicine, Shanghai Shangtishang Orthopaedic Hospital, Shanghai, China

**Keywords:** Parkinson’s disease (PD), pain, exercise, analgesic mechanism, analgesic effect

## Abstract

The research and clinical applications of exercise therapy to the treatment of Parkinson’s disease (PD) are increasing. Pain is among the important symptoms affecting the daily motor function and quality of life of PD patients. This paper reviewed the progress of research on different exercise therapies for the management of pain caused by PD and described the role and mechanism of exercise therapy for pain relief. Aerobic exercise, strength exercise, and mind-body exercise play an effective role in pain management in PD patients. The pain suffered by PD patients is divided into central neuropathic, peripheral neuropathic, and nociceptive pain. Different types of pain may coexist with different mechanistic backgrounds and treatments. The analgesic mechanisms of exercise intervention in PD-induced pain include altered cortical excitability and synaptic plasticity, the attenuation of neuronal apoptosis, and dopaminergic and non-dopaminergic analgesic pathways, as well as the inhibition of oxidative stress. Current studies related to exercise interventions for PD-induced pain suffer from small sample sizes and inadequate research of analgesic mechanisms. The neurophysiological effects of exercise, such as neuroplasticity, attenuation of neuronal apoptosis, and dopaminergic analgesic pathway provide a sound biological mechanism for using exercise in pain management. However, large, well-designed randomized controlled trials with improved methods and reporting are needed to evaluate the long-term efficacy and cost-effectiveness of exercise therapy for PD pain.

## Introduction

Parkinson’s disease (PD) is a progressive neurodegenerative disease and is one of the two most common neurodegenerative diseases in the world; the other one is Alzheimer’s disease (Tandon et al., 2021). PD’s incidence increases with age, and it is most common in the elderly population. The average age of onset is about 60 years old, and PD is rare in young people under 40 years old. It is estimated to affect 9 million people worldwide by 2030 (Dorsey et al., 2007). In PD, dopaminergic and non-dopaminergic neural pathways are affected. The initial lesions of vulnerable nuclear grays and cortical areas cause the loss of dopaminergic neurons in the substantia nigra of the basal ganglia, leading to their dysfunction and affecting dopaminergic neural pathways (Braak et al., 2003). In addition, the degeneration of the dorsal motor nucleus of the vagus and olfactory nuclei damages the lower brainstem, basal ganglia, and forebrain production, and such damage extends to the cortex, thereby affecting the non-dopaminergic neural pathway. The damage to different pathways can also cause different effects and symptoms. When dopaminergic and non-dopaminergic neural pathways are affected, motor and non-motor injuries occur, respectively (Allen et al., 2015). Motor injuries include some bradykinesia, dyskinesia, dystonia, tonic, tremor, and postural instability (Hughes et al., 1992). Non-motor injuries include pain, cognitive and emotional disorders, autonomic dysfunction, sleep disorders, and paresthesia.

Parkinson’s pain is a painful non-motor symptom that affects up to 85% (Jankovic, 2008). The three main types of PD are central neuropathic pain (CNP), peripheral neuropathic pain (PNP), and nociceptive pain. CNP is a type of neuropathic pain caused by a lesion of the somatosensory nervous system, with partial damage to the central nervous system or loss of sensation in the corresponding body area innervated by damaged peripheral nerves (Treede et al., 2008). Most CNPs are due to the disease process itself (Ford, 2010). Its main manifestation is stinging, burning, or ejection pain. PNP is associated with pain caused by radiculopathy or peripheral neuropathy and is restricted to a specific skin or cutaneous nerve distribution, producing sharp, shooting, electric shock, and others (Wasner and Deuschl, 2012). Nociceptive pain includes musculoskeletal, dystonia, visceral, and skin pain (Truini et al., 2013). Musculoskeletal pain is usually caused by an actual or threatening injury to the muscles and joints and may manifest as an injury or dysfunction that causes severe pain during movement. Therefore, the pain felt by PD patients may be related to disease processes, sensitization of the nervous system, and structural or biomechanical abnormalities (Allen et al., 2015). Although pain is among the most troubling symptoms in patients with PD, the recognition and management of pain in clinical practice has received little attention (Nègre-Pagès et al., 2008; Broen et al., 2012). At present, treatment and management of pain in PD patients are based on drugs, including dopaminergic drugs and conventional analgesics that may help reduce pain (Perez-Lloret et al., 2012). Different types of exercise are recommended as components of pain management programs (Domingos et al., 2018; Borisovskaya et al., 2020).

## Effect of exercise for pain management in Parkinson’s disease

European and Canadian clinical guidelines for PD stated that a regular exercise routine that begins early has proven benefits and that exercise therapy should accompany the entire course of PD. The clear benefits of exercise are currently shown in patients with established disease; moreover, exercise performed as early as possible at the time of diagnosis can slow disease progression (Domingos et al., 2018; Grimes et al., 2019). Exercise is effective in improving motor function and cardiovascular health and reducing falls (Bergen et al., 2002; Ashburn et al., 2007; Allen et al., 2011). Pain is a common and poorly treated symptom of PD that affects motor function, depressive status, and daily life (Mostofi et al., 2021). Although pain is not the primary focus of treatment-seeking in PD patients compared with resting tremor, bradykinesia, and dystonia abnormalities, and it is not the primary intervention indicator in clinical studies, exercise-induced hypoalgesia (i.e., an immediate reduction in pain sensitivity following exercise) is reported in people with PD (Nguy et al., 2019).

Exercise therapies that are commonly used in clinical trials to improve pain symptoms in PD patients include aerobic exercise, strength exercises, aquatic Tai Chi training, and flexibility training. Aerobic exercise can produce measurable protection and improvement in PD progression, both physically and cognitively (Schootemeijer et al., 2020). Atan et al. (2019) conducted a randomized controlled trial of body-weight-supported treadmill training (BWSTT) in PD patients and found that BWSTT improved lower limb joint pain in pain domains in the Nottingham health profile (NHP) compared with pre-training and conventional treadmill training. This finding suggested the potential role for load-controlled aerobic walking training in improving bone and joint pain in PD patients. Appropriate strength training can ameliorate musculoskeletal weakness; it can potentially induce adaptive changes in the associated neuromuscular system and reduce the risk of falls in PD patients (Falvo et al., 2008). Rodrigues De Paula et al. (2006) evaluated the impact of a 12-week exercise program on the different quality of life domains in 20 patients with mild to moderate PD. The exercise sessions were performed thrice a week and included strength training and aerobic exercises (stepping and graded walking). An 8% reduction in pain domains was found in NHP, although this finding was not significant. Tai Chi is an increasingly popular mind-body intervention that can treat a variety of motor and non-motor symptoms associated with PD (Song et al., 2017). It combines balance, flexibility, and neuromuscular coordination training with various cognitive components, including increased body awareness and focused mental attention; this combination may confer benefits over and above regular exercise in PD (Wayne et al., 2013). Aquatic Tai Chi training consists of a series of aquatic exercises, based on the Tai Chi concepts (Kurt et al., 2018). Pérez De La Cruz (2017) recruited 30 patients with PD who had a similar disease course and were equally randomly assigned to either the aquatic Tai Chi group or the control group for a 10-week intervention. Using the visual analog scale (VAS), they found that 10 weeks of aquatic Tai Chi training and a conventional exercise routine that focused on gait, balance, and muscle strength reduced the intensity of neuropathic pain in people with PD. Musculoskeletal pain caused by PD is often due to rigidity, dyskinesia, and dystonia. Moreover, flexibility training can reduce joint stiffness, dystonia, and dyskinesia, thereby improving musculoskeletal pain in the trunk and extremities (Wasner and Deuschl, 2012). Reuter et al. (2011) recruited 90 patients with mild to moderate PD who were randomly and equally assigned to three exercise interventions, namely, flexibility and relaxation training, general walking, and Nordic walking. Pain intensity was scored using VAS, and the intensity of neck, hip, and iliosacral pain decreased in nearly one-third of the patients. Such evidence suggested that flexibility and relaxation training may help treat pain arising in the musculoskeletal system in PD patients. Table 1 provides more details of the research on exercise intervention for pain from PD.

**TABLE 1:** Major characteristics of studies focused on exercise for pain management in Parkinson’s disease (PD).

Article, year	Country/ Region	Population sample size study design	Age (years)	Intervention	Duration of trial period	Pain outcomes	Outcome assessment	Result
Rodrigues De Paula et al., 2006	Brazil	20 subjects 1 group M = 14, F = 6	G1(*n* = 20):61.5 y (9.8)	(1) 15-min warm-up; (2) 20-min of strength training; (3) 30-min of aerobic exercises, consisting of stepping and graded walking; (4) a cool-down period.	Three 75-min sessions per week for 12 weeks	Pain domains in NHP	After 12-week intervention	A non-significant 8% reduction in the pain domain of the NHP
Reuter et al., 2011	Germany	90 subjects 3 groups M = 45, F = 45	G1(*n* = 30):62.1 y (2.5) G2(*n* = 30):63 y (3.1) G3(*n* = 30):62 y (3.2)	G1: Flexibility, coordination, and relaxation exercises G2: Walking G3: Nordic walking	Three 70-min sessions per week for 24 weeks	VAS	After 24-week intervention	Up to 30% of patients became free of pain, and the intensity of pain decreased in patients after training
Pérez-De La Cruz et al., 2016	Spain	15 subjects 1 group M = 6, F = 9	G1(*n* = 15):65.87 y (7.09)	G1: Aquatic Tai Chi	Two 30-min to 45-min sessions per week for 10 weeks	VAS	After 10-week intervention at 1-month follow-up	A significant improvement in scores for pain perception
Pérez De La Cruz, 2017	Spain	30 subjects 2 groups M = 13, F = 17	G1(*n* = 15):66.8 y (5.87) G2(*n* = 15):67.53 y (9.89)	G1: Aquatic Tai Chi G2: Therapy on dry land (strength training and aerobic exercises)	Two 45-min sessions per week for 10 weeks	VAS	After 10-week intervention at 1-month follow-up	Both Aquatic Tai Chi and strength training and aerobic exercises reduced pain intensity
Atan et al., 2019	Turkey	30 subjects 3 groups M = 11, F = 19	G1(*n* = 10):69.7 y (8.0) G2(*n* = 10):72.2 y (7.9) G3(*n* = 10):68.6 y (8.2)	G1: control group (unsupported TT) G2: 10% supported BWSTT G3: 20% supported BWSTT	Five 60-min sessions per week for 6 weeks	Pain domains in NHP	After 6-week intervention	Pain subscore decreased in the 10% and 20% supported BWSTT groups after training
Cochen De Cock et al., 2021	France	45 subjects 1 group M = 25, F = 20	G1(*n* = 45):65 y (9.0)	Outdoor, uncontrolled gait rehabilitation program using the BeatWalk application	Five 30-min sessions per week for 4 weeks	Pain with visual sliding scales	After 4-week intervention	During BeatWalk use, the patients reported a significant reduction of pain relative to the baseline

M, male; F, female; G, group; y, year; NHP, Nottingham health profile; VAS, visual analog scale; TT, treadmill training; BWSTT, body-weight-supported treadmill training.

## Mechanisms of exercise for pain management in disease

**FIGURE 1:**
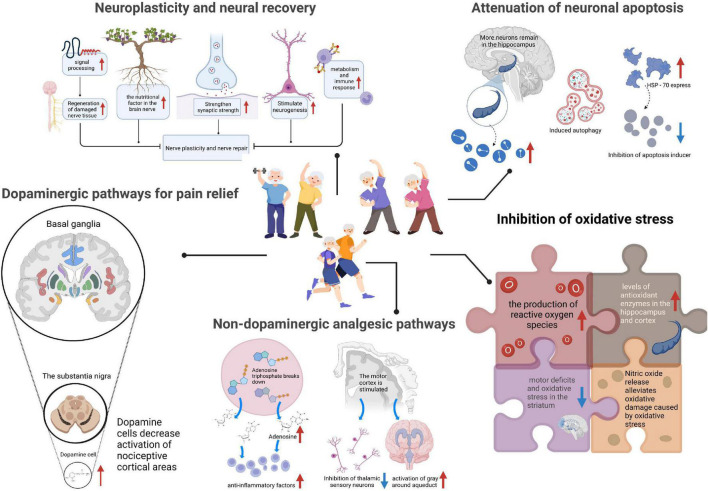
Mechanisms of exercise for pain management in Parkinson’s disease (PD). The analgesic mechanism of exercise on PD-induced pain, including neuroplasticity and neural recovery, attenuation of neuronal apoptosis, dopaminergic analgesic pathway, non-dopaminergic analgesic pathway, and inhibition of oxidative stress. HSP-70, the 70 kilodalton heat shock proteins.

The pain suffered by PD patients is divided into central, peripheral, and nociceptive pain. Both central and peripheral pain are neuropathic (Wasner and Deuschl, 2012). The different types of pain may coexist with different mechanistic backgrounds and treatments. Nociceptive pain involves the trunk and lower back. Neuropathic pain is more common in the upper and lower limbs. For pain evaluation, studies using electroencephalogram positioning pain assessment matrix observation found in the condition of high pain, before and after the thalamus, somatosensory cortex, insula, medial, and lateral prefrontal cortex that cingulate structure frequency specificity of neuron activity increased, and the activation of pain relief in these regions was significantly reduced (Prichep et al., 2011). Therefore, the activity of neurons in these areas is the main indicator of pain. At present, several mechanisms by which exercise improves PD-induced pain exist, including neuroplasticity and neural recovery, attenuation of neuronal apoptosis, dopaminergic analgesic pathway, non-dopaminergic analgesic pathway, and inhibition of oxidative stress (Figure 1).

### Neuroplasticity and neural recovery

Exercise can improve the underlying mechanism of signal processing to regenerate the damaged nerve tissue of PD patients and promote physiological and functional reorganization to adapt to environmental changes (Petzinger et al., 2013). Specifically, exercise can increase the levels of brain-derived neurotrophic factor (BDNF) and induce changes in dendritic spines (Mattson, 2014). There is little evidence that progressive resistance exercise, dancing, and high-tempo cycling can improve dyskinesia and relieve pain through exercise. Using transcranial magnetic stimulation and the research method of measurement of changes in the excitability of cortex movement in sports training, the experiment randomly assigned participants to high strength, low intensity, and zero intensity treadmill training. Eight weeks of moderate to severe intensity treadmill training can enhance the function of the cortex and basal ganglia ring and improve the activity. Participants in the study showed significant improvements in stride speed, swing, and postural time, especially in the high-intensity group (Lefaucheur, 2005). In the measured results, the silent period of the cortex was shortened, indicating an increased level of motor system excitability. An animal study showed that dendritic spinal injury in a rodent model of PD was reversed by running on a treadmill (Toy et al., 2014). Further studies in rodent models of diabetes showed that neuropathic pain might be caused by the remodeling of maladaptive dendritic spines (Tan et al., 2012). Exercise could induce changes in dendritic spines that reduced pain in PD patients. In addition, voluntary exercise could increase BDNF levels (Galvan and Wichmann, 2007). BDNF has neuroprotective and neurotrophic properties; it can enhance brain plasticity and promote synapses and neurogenesis to strengthen neurons and mediate the connection between movement and the brain (Cotman and Berchtold, 2002; Cotman et al., 2007). As BDNF increases, nerve recovery becomes more rapid and robust, thereby helping relieve pain. However, it is not clear from this study how long these changes persist after exercise needs to be further explored. In conclusion, neuroplasticity and neural recovery are among the mechanisms by which exercise improves pain in PD patients.

### The damping of neuronal apoptosis

Apoptosis is programmed cell death; its features are cellular swelling, film rupture, and random degradation of DNA (Zhu et al., 2022). There are three kinds of apoptosis pathways, which are the endoplasmic reticulum, receptor, and chondriosome pathways (Park et al., 2021). The effect of exercise on apoptosis involves the DNA degradation of chromatin, DNA fragment formation, cytoplasmic foam, and apoptotic body. Among them, the study of the apoptotic body is more detailed. At present, the main cause of PD is the apoptosis of dopamine neurons in the substantia nigra (Braak and Del Tredici, 2008). The method of inhibiting neuronal apoptosis led to better results. Exercise alleviates tissue damage caused by cerebral ischemia, prompting the retention of more surviving neurons in the hippocampus and effectively reducing neuronal death. Physical exercise can play a role in protecting nerves in the clinic, promoting angiogenesis and modulating inflammatory reactions (Eadie et al., 2005). Some studies explored the influence of physical exercise on cell apoptosis and found that exercise could effectively induce cell autophagy and heat shock protein (HSP-70) expression, thus inhibiting apoptosis-inducing factors, increasing the expression of anti-apoptotic proteins, and further attenuating apoptosis (Trejo et al., 2001; Zhang et al., 2011). In addition, the neuroprotective effects of various frequencies on neuronal apoptosis have been studied; performing high strength preconditioning exercise three or more times a week can potentially alleviate the problem of apoptosis and the production of anti-apoptosis-related proteins through preconditioning and other methods. Therefore, future studies need to explore the appropriate exercise intensity, frequency, and time points to better solve the relevant problems.

### Dopaminergic pathways for pain relief

The deficiency of folic acid in PD patients increases the level of homocysteine, which damages the DNA of nerve cells in the substantia nigra, affects the production of dopamine, and leads to the dysfunction of nerve cells, thereby resulting in pain. This pathway relies on dopamine-producing cells in the substantia nigra of the basal ganglia to influence pain (Olanow and Schapira, 2013). It inhibits the conduction of nociceptive signals in the dorsal root ganglia and regulates pain through the ventral medulla (Barceló et al., 2012; Galbavy et al., 2013). In a trial that involved 8 weeks of moderate to vigorous treadmill training in patients with PD, the binding potential of dopamine D2 receptors in the striatum increased in patients with PD, suggesting that exercise increases dopamine receptor binding capacity, thereby inhibiting nociceptive and alleviating pain (Fisher et al., 2013). Dopaminergic transmission inhibits nociception and regulates pain. In general, dopaminergic drug use is the most common intervention. Levodopa significantly increased the pain threshold in painless PD patients (Gerdelat-Mas et al., 2007). Positron emission scans, which looked at neuroimaging results during experimental pain stimuli, showed that the hyperactivation of several injury-causing cortical regions in PD patients was reduced by the use of levodopa (Brefel-Courbon et al., 2005). Although the results showed that the objective pain threshold of PD patients was generally lower than that of healthy people, the administration of levodopa increased the objective pain threshold of PD patients and verified the participation of the dopaminergic system (Djaldetti et al., 2004). Pain may be accompanied by other related symptoms, including emotional, cognitive, and reactive levels, due to the involvement of the dopaminergic system. PD patients with pain tend to have more severe depression, and there is a strong correlation between pain and depression (Ehrt et al., 2009). The absence of norepinephrine, dopamine, and other substances in the body can lead to depression. Older patients are at risk of falling. Therefore, the dopaminergic pathway can alleviate the occurrence of other complications while improving pain.

Glutamate is the main excitatory neurotransmitter in the brain (Bleakman et al., 2006). Glutamatergic conduction is closely related to the sensitivity of the central nervous system, which can produce hypersensitive responses to harmful or non-harmful information. Exercise improves dopamine transmission and affects glutamate transmission and availability, thereby improving basal ganglia function (Petzinger et al., 2013).

### Non-dopaminergic analgesic pathways

It has been hypothesized that PD pain is caused by neuronal cell loss, formation of Lewy bodies in parabrachial regions, and changes in the periaqueductal gray matter of the spinothalamic pain pathway, which affect affective, cognitive, and autonomic responses (Ford, 2010). However, the effect of exercise on non-dopamine structures is less clear. The breakdown of adenosine triphosphate, which is released during exercise, improves pain primarily by increasing extracellular adenosine (Dworak et al., 2007; Roque et al., 2011). Adenosine has an anti-inflammatory effect, can inhibit the expression of pro-inflammatory cytokines, and increase the expression of anti-inflammatory cytokines. Exercise can stimulate the motor cortex, leading to the inhibition of thalamic sensory neurons and increased activation of the periaqueductal gray, thereby integrating the received nociceptive input to regulate pain (Pagano et al., 2012). According to the study, the inhibition of aerobic exercise is a cause of pain, and the hypersensitivity response is significantly reduced after repeated exercise. Reduced hypersensitivity stabilizes the disordered parts of the body, improving its ability to deal with pain. Another study specifically focused on exercise therapy for PD patients with back pain; the initial evaluation was followed by exercise in the supine position (Rosarion, 2018). In the fifth week of a new exercise session, the patient reported improved exercise recovery and endurance compared with previous training sessions. According to the process and results, exercise can improve bradykinesia and the maintenance of postural balance, as well as enhance the quality of life of patients. During exercise, muscle contractions help strengthen muscles, giving the patient more motor control.

### Inhibition of oxidative stress

Another important cause of pain is oxidative stress and free radical damage. Iron produces a large number of toxic hydroxyl radicals in the REDOX process. The nerve cells of the nigra and striatum are highly sensitive to oxidative stress and are easily damaged by accumulated iron. When the body is stimulated, oxidative damage to the mitochondria leads to the increased release of pro-apoptotic mediators, which disrupts the oxidation antioxidant balance and leads to the damage of the oxidation organization (Lushchak et al., 2021). Exercise can promote the production of reactive oxygen species in cells, further eliminating them to maintain body homeostasis (Zhu et al., 2022). Meanwhile, long or short-term pretreatment can improve the levels of antioxidant enzymes in the hippocampus and cortex and inhibit some oxidative stress. When performing physical exercise to prevent cerebral ischemia-reperfusion movement disorders and imbalances in the study of striatum oxidation, the 8-week intervention plan included placing rats on an electric treadmill. Movement disorder was found to be caused by the damage to the striatum; in neurodegenerative diseases, the oxidation of striatum causes imbalance and loss of dopaminergic neurons and neurotoxicity (Brooks and White, 1978; Park et al., 2013). Exercise can effectively avoid or minimize motor defects and oxidative stress in the striatum. Specific types of physical exercise, such as swimming training, inhibit glutamate and promote the release of nitric oxide, which alleviates oxidative damage caused by metabolic stress.

## Conclusion

Pain greatly affects daily quality of life of PD patients. It may affect normal walking, going up and down stairs, perception, cognition, and sleep emotion. Therefore, improving pain in PD patients is crucial. Other co-morbidities, such as depression and dyskinesia, should also be treated so that patients can have a good quality of life. Although many studies investigated the improvement of symptoms and quality of life with exercise, few studies focused on the pain relief of patients by means of exercise. High-quality large sample randomized controlled trials are lacking.

At present, most of the studies on the mechanism of exercise to improve pain have expounded on the improvement methods of exercise for pain, which can be achieved through neural recovery, dopamine, non-dopamine, inhibition of oxidative stress, and other ways. However, some shortcomings and questions still need to be addressed, such as how long the improvement in pain will last, as well as the appropriate intensity, frequency, and timing of exercise. In addition, the research volume in the current study is small, and the period is short. So, some interfering factors may not be excluded. In future studies, research needs to focus on specific details on the discussion of the effects and benefits of exercise sustainability, as well as some details on pain relief exercises and data to help symptomatic pain relief.

## Author contributions

X-QW conceived the review and revised the table in the drafted manuscript. W-YY and Q-HY drafted the manuscript, searched the literature to identify eligible trials, and extracted and analyzed the data. All authors approved the final manuscript.
